# Effects of Dietary Quinoa Seeds on Cecal Microorganisms and Muscle Fatty Acids of Female Luhua Chickens

**DOI:** 10.3390/ani12233334

**Published:** 2022-11-28

**Authors:** Tao Wu, Xiaofan Jiang, Farong Yang, Yuming Wei, Shengguo Zhao, Ting Jiao

**Affiliations:** 1 College of Animal Science and Technology, Gansu Agricultural University, Lanzhou 730070, China; 2 Animal Husbandry Pasture and Green Agriculture Institute of Gansu Academy of Agricultural Sciences, Lanzhou 730070, China; 3 College of Grassland Science, Gansu Agricultural University, Lanzhou 730070, China

**Keywords:** Luhua chickens, Chenopodium quinoa, fatty acid, 16S rRNA, microorganisms

## Abstract

**Simple Summary:**

Providing high-quality feed for animals to improve the quality of animal products and, therefore, meet the needs of the public is a focus of animal husbandry. As a high-altitude crop, quinoa has high and abundant nutritional value. In recent years, with the planting area increasing year by year in our country, feed development and utilization of quinoa seeds and by-products have increased. At present, few studies gave examined the optimal addition of quinoa seeds in livestock and poultry diets and, in particular, the effects of the dietary addition of quinoa seeds on the intestinal microorganisms and muscle fatty acids of Luhua chickens. Therefore, in this study, different proportions of quinoa seeds (raw grain) were added in the diets of Luhua chickens to analyze their effects on intestinal microbiota and muscle fatty acids. The results showed that the content of muscle docosahexaenoic acids (C22:6n3, DHA), unsaturated fatty acids (UFAs) and n-3 polyunsaturated fatty acids (n-3 PUFAs) was significantly increased by adding a certain proportion of quinoa seeds to the diet. The content of saturated fatty acids (SAFs) and the ratio of n-6/n-3 significantly decreased. The increase can effectively improve the cecal microbiota structure, regulate the intestinal environment and promote the body health of Luhua chickens. These data provide a theoretical basis for the scientific application of quinoa seeds in female Luhua chickens’ feeding and fodder development.

**Abstract:**

To study the effects of adding quinoa seed (raw grain) to the diet of the Luhua chicken on the cecal microorganism abundance and fatty acid composition of muscle, 120 49-day-old healthy female dewormed Luhua chickens (body weight 1476.21 ± 101.39 g) were randomly divided into 4 groups, with 3 replicates in each group and 10 chickens in each repetition. The control group (CK group) was fed a basal diet and the experimental groups were fed with 4% (Q4), 8% (Q8), and 12% (Q12) quinoa seed (raw grain) added to the basal diet for 75 days. After 121 days of age, the animals were slaughtered and the 16S rRNA characteristics of cecal flora, as well as composition and content of fatty acids in muscle, were determined and analyzed. The content of unsaturated fatty acids (UFAs), docosahexaenoic acid (C22:6n3; DHA) and n-3 polyunsaturated fatty acids (n-3 PUFAs) in the breast and leg muscles significantly increased in the experimental groups supplemented with quinoa seeds (*p* < 0.05). However, the content of saturated fatty acids (SAFs) and ratio of n-6/n-3 in breast muscle and leg muscle significantly decreased (*p* < 0.05). In addition, adding a certain percentage of quinoa seeds in the diet can also affect the community composition and content of microorganisms in the ceca of Luhua chickens. At the phylum level, the *Proteobacteria*, *Actinobacteria*, *Synergistetes* and *Melainabacteria* in experimental groups (Q4, Q8 and Q12) were significantly lower than those in the CK group (*p* < 0.05). At the genus level, *Desulfovibrio*, *Synergistes*, *Olsenella*, *Parabacteroides*, *Mailhella*, *Sutterella* and *Ruminiclostridiu* in group Q4 were significantly lower than those in group CK (*p* < 0.05) while *Faecalibacterium* in Q8 group, and *Lawsonia* and *Faecalibacterium* in Q12 group were significantly higher than those in the CK group (*p* < 0.05). Enrichment analysis of the microbial function showed that compared with the CK group, *Metabolism* and *Enzyme Families* were significantly enriched in the Q4 group (*p* < 0.05). *Cellular Processes and Signaling* were significantly enriched in the Q8 group (*p* < 0.05). The association analysis of fatty acids with microorganisms showed that the abundance of *Faecalibacterium*, *Lawsonia* and *Meagmonas* was significantly correlated with partial SFAs and UFAs (*p* < 0.05). In conclusion, adding quinoa seeds to diets significantly increased the content of muscle DHA, UFAs and n-3 PUFAs. The content of SAFs and the n-6/n-3 ratio were significantly reduced. Taken together, quinoa can effectively improve the cecal microbiota structure, inhibit the number of harmful bacteria and increase the number of beneficial bacteria, regulating the intestinal environment and promoting the body health of female Luhua chickens.

## 1. Introduction

Chenopodium quinoa is an annual dicotyledonous herb of the genus Chenopodiaceae, which originated in the Andes Mountains of South America and has been cultivated for more than 5000 years. Compared with other crops, quinoa has strong adaptability and can grow in high altitude, dry and cold areas [1]. Because quinoa seeds are rich in protein, minerals, vitamins, amino acids and other nutrients [2,3,4], including essential amino acids, the National Aeronautics and Space Administration (NASA) regards quinoa seeds as the ideal “space food” [5,6]. Saponins in quinoa have antibacterial, antioxidant, immunomodulatory, anti-tumor and other pharmacological regulatory effects [7]; and its straw, seed and bran all have great feeding potential [8,9]. Quinoa seeds can provide minerals, protein and vitamins for livestock, and improve the proportion of amino acids in feed. Byproducts, such as quinoa straw, bran and chaff, show comparable feeding value to other superior forage materials in feeding livestock [8,9]. The nutritional value of the quinoa whole plant and its straw is comparable to that of corn, alfalfa and sweet sorghum, with high digestibility [8]. The lignin content of quinoa straw is lower than that of corn straw, but is suitable for the production of roughage for ruminants such as cattle and sheep [8]. Currently, quinoa research is focused onbreeding, cultivation and primary processing. There are few studies on the utilization of quinoa seeds and their byproducts.

Currently, intestinal microorganisms are receiving increasing attention due to their vital role in intestinal development and metabolic homeostasis [10]. Studies have found that mammalian gut microbiota is closely related and potentially important to food digestion, vitamin and amino acid synthesis [11], organ development [12,13], host physiological regulation [14], immune system regulation, and growth and neural development [15,16]. Some studies have found that adding a certain amount of washed and cooked quinoa seeds in the diet has no significant effect on the performance of chickens [17]. As the most important intestinal segment in the distal part of the chicken gut, the ceca of chicken is a complex ecosystem with the highest concentration of microorganisms and highly diversified microbiota in adult chickens, which has an important impact on the growth and development of chickens [18,19,20]. The digestion and absorption of nutrients in cecum are closely related to its microorganisms [21]. Ceca resection could significantly reduce the digestibility and metabolism of nutrients in poultry [22]. The analysis of ceca microbiota was a key field in poultry nutrition research, which was helpful to understand its diversity and interaction with the host [23]. Microorganisms in the ceca could transform fiber components into digestible components through fermentation, thus promoting animal growth. Thus, it is necessary to study the intestinal microbial diversity of the Luhua chicken. Intramuscular fat deposition was mainly affected by animal age, sex, breed, and nutrition level. In recent years, more and more studies have confirmed that intestinal flora could also regulate intramuscular fat deposition in animals [24,25]. One study found that intestinal microbes could regulate fat metabolism and affect the level of fat deposition in blood and tissues [26,27,28,29]. Intramuscular fat deposition in animals is also regulated by intestinal flora [24,25,30,31]. Relevant studies have shown that the flora in duodenum and the ceca of broilers have a great influence on fat deposition, and the abundance of *Methanobacterium* and *Mucispirillum Schaedleri* in ceca have a significant positive and negative correlation with abdominal fat deposition, respectively [32]. The fat deposition of broilers can be affected by regulating intestinal flora. Intestinal microorganisms play an important role in the process of fat digestion, absorption and deposition in animals, and further affect intramuscular fat deposition.

With the improvement in living standards, people have higher and higher expectations of meat quality. High quality, healthy and safe chicken meat is favored by consumers. Studies have shown that a high concentration of n-3 PUFAs, increased concentration ratio of unsaturated fatty acids (PUFA/SFA) and n-3/n-6 are beneficial to human health [33]. Since the intake of n-3 PUFAs in the human body is low [34], scientists have shown great interest in producing chickens rich in n-3 PUFAs for human consumption [35]. Marino R et al. [36] found in their study that due to the close connection between stress and the immune system, supplementing flaxseed and quinoa seeds can reduce the impact of stress on animals and significantly improve muscle tenderness.

So far, no studies have evaluated the effects of dietary quinoa seeds on intestinal microbiota and muscle fatty acids of Luhua chickens. Therefore, this study used 16S rRNA microbiome and gas chromatography to evaluate the effects of different formulations made with quinoa seeds as feed materials on gut microbes and muscle fatty acids of Luhua chickens. Correlation analysis between gut microbes and fatty acids may provide a theoretical basis for exploring the mechanism of quinoa seeds on cecal microbiota and fatty acid deposition of broilers and its application in Luhua chicken production.

## 2. Materials and Methods

### 2.1. Moral Statement

All procedures for the use of laboratory animals were approved by the Laboratory Animal Management Committee of Gansu Agricultural University under license number GSAU-2019-0018, and the animals were tested in accordance with the guidelines of the committee. Experiments involving animals shall be conducted in accordance with the Regulations on the Administration of Laboratory Animals (Ministry of Science and Technology of China. Revised June 2004). Samples were collected in accordance with the Guidelines of the Ethics Committee for the Care and Use of Laboratory Animals of Gansu Agricultural University.

### 2.2. Experimental Design

In this experiment, a single factor completely random design was adopted. A total of 120 dewormed healthy female Luhua chickens (weight 1476.21 ± 101.39 g) aged 49 days were selected and randomly divided into 4 groups, with 3 replicates in each group and 10 chickens in each repetition. Hens in the control group (CK group) were fed a basal diet, and hens in the experimental groups were fed the basal diet supplemented with 4% (Q4), 8% (Q8) and 12% (Q12) quinoa seeds (original diet). The experimental period lasted for 75 days, including the pre-experimental period of 3 days and the formal experimental period of 72 days.

### 2.3. Experimental Diet and Feeding Management

#### 2.3.1. Composition and Nutritional Level of Experimental Diets

The diets of the experimental chickens were formulated according to the nutritional requirements of broilers in “Chicken Feeding Standards (NY/T33-2004)” and, combined with the Luhua chicken breeding manual, the nutritional levels of the experimental chickens all met the nutritional requirements of Luhua chickens. The composition and nutritional levels of the experimental basal diets are shown in Table 1.

#### 2.3.2. Test Animals

Forty-nine-day-old healthy dewormed Luhua chickens were purchased from Longsheng Family Farm, Beizhai Town, Weiyuan County. Quinoa seeds were provided by the Tianzhu Quinoa Alpine Test Station of the Gansu Academy of Agricultural Sciences. The basal diet was produced by Gansu Aonong Feed Science Technology Co., Ltd (Wuwei, China), according to the experimental formula.

#### 2.3.3. Experimental Animal Feeding and Management

The feeding experiment was carried out in the training base of the Animal Science and Technology College of Gansu Agricultural University. Ten days before the experiment, the coop was thoroughly cleaned and washed, utensils such as the sink and trough were cleaned, and walls were also disinfected with lime water. After cleaning and drying, the doors and windows of the henhouse and ventilation fan were sealed and the whole henhouse was fumigated with potassium permanganate for 24 h before the doors and windows were opened for ventilation for 1 week. The coop was kept dry, clean and well-ventilated. The chickens were examined, immunized, disinfected, numbered and weighed. The chicken crates from the farm we use were sterilized before being transferred into the henhouse (powder, water spray) to help maintain disinfection. During the breeding period, the coop (with chickens) was disinfected once every 2 weeks with sanitizer or 0.1% new germicide. Waste was regularly cleaned from the henhouse as much as possible and steps were taken to avoid inducing various chicken diseases. During the experiment, the chickens were fed twice at a fixed time (7:00 and 15:00 p.m.) every day and free to sufficiently eat and drink. Sufficient feed was added to each trough to ensure feed was in the trough at all times while the chickens were feeding. The diet was provided as mash during the whole experimental period from 12 July 2019 to 26 September 2019. During the rearing process of the broilers, the environmental indicators of the hen house were strictly controlled and recorded. The control range of the main indicators were as follows: temperature 22–28 °C, humidity 60–65%, light time controlled at about 16 h (light on from 6:00 a.m. to dawn, light on at 18:00 p.m. and light off at 22:00 p.m.); and the coop was divided into three layers: upper, middle and lower.

### 2.4. Collection and Processing of Test Samples

#### 2.4.1. Collection and Processing of Muscle Tissue Samples

On the 75th day of the experiment, two Luhua chickens were randomly selected from the different replicates of each group. After weighing, the chickens were sacrificed by cervical dislocation and bled, feathers were removed; and after draining water, the slaughter performance index was determined. Breast muscle and leg muscle samples with skin and fascia removed were collected and vacuum packed and placed in the refrigerator at −80 ℃ for testing.

#### 2.4.2. Collection of Cecal Microbial Samples

At the age of 121 days, two Luhua chickens were randomly selected from each group of the different replicates and slaughtered. Sterilized surgical scissors were used to separate the ceca from other different intestinal segments, and the cecal contents were collected into 5 mL cryopreserved tubes, quickly put into a liquid nitrogen tank, and stored in the −80 ℃ cryogenic refrigerator measuring intestinal microbial diversity.

### 2.5. Determination of Indexes and Methods

#### 2.5.1. Determination of Fatty Acid Content in Muscle

The meat samples in the vacuum packaging bag were thawed at room temperature for 12 h, and the muscle surface was peeled off and removed with a knife. The samples were placed in a mortar and ground with liquid nitrogen, and then 1.0 g of the ground samples were weighed in a 10 mL stopper tube. Then, 0.7 mL of KOH solution at a concentration of 10 mol·L^−1^ and 5.3 mL of anhydrous methanol (analytical methanol (for chromatography)) were added, respectively, and the test tube was shaken for 5 s every 20 min in a water bath at 55 °C for 1.5 h. At the end of the water bath, the tube was removed and cooled to below room temperature under tap water. Then, 0.58 mL of 12 mol∙L^−1^ H_2_SO_4_ solution was added, and the constant temperature water bath was continued at 55 °C for 1.5 h for free fatty acid methylation, while the tube was shaken every 20 min for 5 s. At the end of the water bath, the test tube was cooled with tap water to below room temperature, 3 mL n-hexane was added, the tube shaken well, then transferred to the centrifuge tube, centrifuged at 3000 r·min^−1^ for 5 min. The supernatant was filtered into the sample bottle using the organic phase filtration membrane, heated at 45 °C, and applied to a concentrate 2 mL sample to less than 1.5 mL. Samples were stored at −20 °C for gas chromatography (GC) detection.

Three parallel samples were tested for each sample and the average value of the three samples was taken as the test result of the sample. Fatty acids were identified according to the relative retention times of fatty acid methyl ester standards. The peak area normalization method was used to quantify the fatty acid. The ratio of PUFA/SFA to n-6/n-3 was calculated according to the composition and specific content of the measured fatty acids.

##### Chromatographic Condition

The contents of fatty acids in muscle were determined by gas chromatography (GC-2010 plus; Shimadzu Company, Kyoto, Japan). The chromatographic column was an SPTM-2560 capillary column (100 m × 0.25 mm × 0.2 m), and the injection volume was 1.0 μL. Chromatographic conditions: the temperature of the injector detector was 250 °C, nitrogen flow rate 1.11 mL·min^−1^, and split ratio 100:1. Programmed heating mode: the initial temperature was kept at 100 °C for 5 min, and then the temperature was increased to 240 °C at 4 °C∙min^−1^ for 30 min, with a total time of 70 min.

#### 2.5.2. Genomic DNA Extraction, PCR Amplification and 16S rRNA High-Throughput Sequencing

##### Genomic DNA Extraction

Cecal content was collected from 6 chickens in each group for gut microbiome analysis. Genomic DNA was extracted by the CTAB method, and its concentration and purity were detected by a Nanodrop 2000 ultrafine spectrophotometer. After that, agarose gel electrophoresis was used for detection. An appropriate amount of DNA was placed into a centrifuge tube, and the sample was diluted to 1 ng·μL^−1^ with sterile water. DNA samples were stored at −80 ℃ in an ultra-low temperature refrigerator for later use.

##### PCR Amplification

Using diluted genomic DNA as a template, the characteristics of cecal microbiota were obtained by amplification of the V4 region of the 16S rRNA gene, according to the selection of the sequencing region. Subsequently, the 16S V4 region was selected and specific primers with barcode (515F and 806R) were used to identify the bacterial diversity. The specific primers were: 515F (5′-GTGCCAGCMGCCGCGGTAA-3′), 806R (5′-GGACTACHVGGGTWTCTAAT-3′). PCR was performed using New England Biolabs’ Phusion High-Fidelity PCR Master Mix with a GC Buffer, and high-performance High-Fidelity enzymes to ensure amplification efficiency and accuracy.

Reaction procedure: pre-denaturation at 95 °C for 5 min, 25 cycles (denaturation at 95 °C for 30 s. Annealing at 55 °C for 30 s. 72 °C extension 40 s). Extend at 72 °C for 7 min.

##### Mixing and Purification of PCR Products

The PCR products were detected by electrophoresis using 2% agarose gel, and the same amount of samples was mixed according to the concentration of PCR products, and the PCR products were detected by electrophoresis using 2% agarose gel after full mixing, for the target strip. The adhesive recovery kit provided by Qiagen Company was used to recover the product.

##### Library Construction and Computer Sequencing

The TruSeq^®^ DNA PCR-Free Sample Preparation Kit was used for library construction. The constructed libraries were quantified by Qubit and Q-PCR. After the qualified libraries were tested, NovaSeq6000 (Illumina, San Diego, CA, USA) was used to finish the sequencing.

#### 2.5.3. Bioinformatics Analysis

The data obtained from sequencing on the Illumina NovaSeq platform were spliced with various sample reads using FLASH (V1.2.7) [37] to obtain Raw Tags; then, after strict filtering [38], the Tags data (Clean Tags) were obtained. The Tags were then compared with the species annotation database to remove the chimera sequences and obtain Effective Tags [39]. The Effective Tags were clustered by operational taxonomic units (OTUs) with 97% similarity using Usearch software (Uparse v7.0.1001). OTUs were annotated based on the Silva (bacterial) taxonomy database. Based on the results of the OTUs analysis, the samples were analyzed at each taxonomic level, and the community composition of each sample was obtained at the taxonomic level of kingdom, phylum, class, order, family, genus and species. R software (Version 2.15.3) was used for alpha diversity analysis and beta diversity index difference analysis between groups to obtain the Alpha diversity index with the Ace, Chao1, Shannon and Simpson indices and sample dilution curve. PICRUST was used for the functional prediction of microbial communities in the samples [40,41,42].

#### 2.5.4. Statistical Analysis

All statistical analyses were performed with SPSS 22.0 software (SPSS Inc., Chicago, IL, USA). The differences in fatty acid composition between breast and leg muscles were analyzed, and the results were expressed as mean ± standard deviation. *p* < 0.05 was statistically significant, *p* < 0.01 was extremely statistically significant. The Spearman correlation test was used to analyze the correlation between the composition and content of fatty acids in muscle of the breast and leg and cecal microbes at the genus level (relative abundance > 0.5%).

## 3. Results

### 3.1. Effect of Quinoa on Fatty Acid Composition and Content in Breast Muscle of Female Luhua Chicken

Twenty-three fatty acids were detected in the breast muscles of each group, including eight SFAs, 7 species of MUFAs and 8 species of PUFAs (Table 2). The content of C16:0, C16:1, C18:0, C18:1n9c, C18:2n6c, C20:4n6 and C24:1 was higher than 1% in each group. The content of C18:1n9c was the highest among the groups, about 30% among the groups; followed by C16:0, about 25% among the groups; and C18:2n6c was the third highest among the groups; its content was about 12% in all groups. Compared with the CK group, C14:0, C16:0, C17:0, C18:0, C18:2n6c, C18:3n3, SFAs, MUFAs, n-6/n-3 and n-6 PUFAs in the experimental groups (Q4, Q8 and Q12 groups) were significantly lower (*p* < 0.05). C16:1, C17:1, C18:1n9c, C18:1n9t, C20:1, C20:2, C20:3n6, C22:6n3 (DHA), C24:1, UFAs and n-3 PUFAs in the experimental groups were significantly higher than those in the CK group (*p* < 0.05). The content of C4:0 and C8:0 in the CK group was significantly higher than those in the Q8 and Q12 groups (*p* < 0.05), content of C14:0 in the Q12 group was significantly lower than those in the CK, Q4 and Q8 groups (*p* < 0.05), and content of C20:3n6 in the Q4 group was significantly higher than those in the CK, Q8 and Q12 groups (*p* < 0.05). However, there were no significant differences in C15:0, C18:3n6, C20:4n6 (AA), C22:0, PUFAs or PUFA/SFA between the experimental groups and CK group (*p* > 0.05).

### 3.2. Effect of Quinoa on Fatty Acid Composition and Content in Leg Muscle of Female Luhua Chicken

Twenty-six fatty acids were detected in the muscles of each experiment group, including 9 kinds of SFAs and 17 kinds of UFAs; among the UFAs, there were 8 MUFAs and 9 PUFAs (Table 3). The content of C16:0, C16:1, C18:0, C18:3n3 and C20:4n6 (AA) was higher than 1%, and C18:1n9c was the highest, with the content of about 33% among all groups. The content of C16:0 in each group was about 26%. The third highest content was C18:2n6c, which was about 14% among the groups. There are significant differences in some fatty acids of leg muscle in diets supplemented with a certain amount of quinoa, among which, C14:1, C15:1, C16:1, C17:1, C18:1n9c, C18:1n9t, C18:3n3, C18:3n6, C20:1, C20:5n3 (EPA), C22:6n3 (DHA), C24:1, UFAs, MUFAs, n-3 PUFAs and the ratio of PUFA/SFA, Q4, Q8, Q12 were significantly higher than those in the CK group (*p* < 0.05). The content of C14:0, C16:0, C17:0, C18:0, C18:2n6t, C21:0, SFAs and n-6/n-3 ratio in the experimental groups (Q4, Q8, Q12) was significantly lower than those in the CK group (*p* < 0.05). The content of C18:2n6c and EPA in the Q4 group was significantly higher than those in the CK, Q8 and Q12 groups (*p* < 0.05). The content of PUFAs in the Q8 group was significantly higher than that in the CK group (*p* < 0.05). However, there were no significant differences in C12:0, C13:0, C15:0 or n-6 PUFAs between the experimental groups and CK group (*p* > 0.05).

### 3.3. Cecal Microbial Diversity

**Figure 1:**
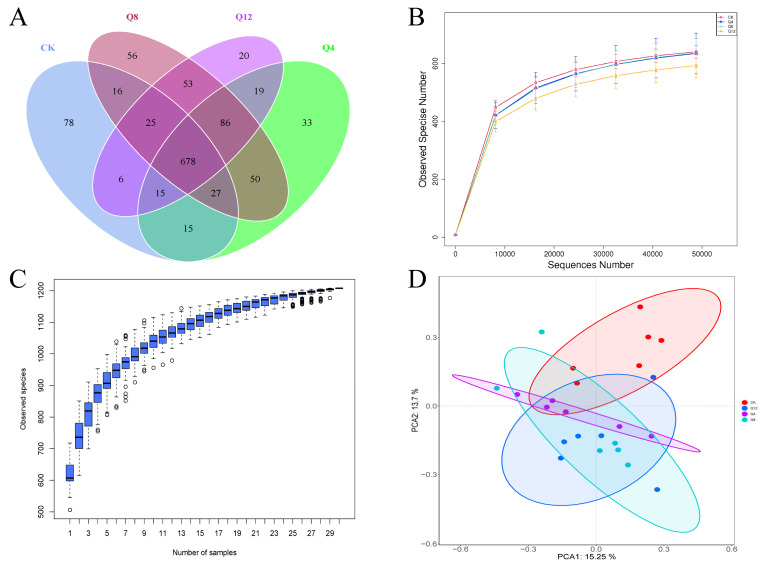
Analysis of cecal microbial diversity. Notes: (**A**) Operational taxonomic units−Venn (OTU−Venn) analysis; (**B**) Saturation curve of species number (dilution curve); (**C**) Species accumulation box pattern; (**D**) PCA analysis.

The PCR-free library was constructed based on the Illumina Nova sequencing platform, and paired-end sequencing was performed. By splicing Reads, an average of 90,178 tags were obtained per sample, and an average of 85,917 tags were obtained by quality control. The effective data amount of quality control was 65,201, and the effective rate of quality control was 72.34% (Table 4). Using Uparse software, Effective Tags were clustered at a 97% similarity level, and the number of OTUs samples was obtained. A total of 1209 OTUs were obtained; there were 850, 923, 991 and 902 OTUs in CK, Q4, Q8 and Q12 groups, and 78, 33, 56 and 20 unique OTUs in CK, Q4, Q8 and Q12 groups, respectively. The total number of OTUs shared by the 4 groups was 678 (Figure 1A). Then, the OTUs sequence and Silva132 database were annotated. A total of 484 OTUs (40.03%) were annotated to the genus level. The dilution curve described the species diversity and species richness of the samples, and tended to flatten at 40,000 reads, indicating that the sequence coverage was saturated. This indicates that the species evenness in the samples tends to stabilize under the experimental conditions, and that the current sequencing depth of each sample is sufficient to reflect the microbial diversity contained in the community sample (Figure 1B). As shown in Table 5, the Alpha Diversity Index showed that Shannon was significantly higher in the CK group than in each Test group, and the Simpson index of the CK group was significantly higher than the Q4 group, indicating that cecal microbial species abundance was significantly higher in the CK group than in each test group. The plot of species accumulation (Figure 1C) shows that the number of species and common species in the environment has reached saturation with the increasing sample size but does not increase with the increasing sample size. As shown by the PCA figure (Figure 1D), there were significant differences in the cecal microbial species between the CK group and trial group with different proportions of quinoa added, as well as some differences between the different proportions of quinoa added.

### 3.4. Cecal Microbial Composition

By comparing with the Silva132 database (Table 6), the species were annotated, and the statistical results of different taxonomic levels were as follows: There were a total of 1209 OTUs, among which the number of OTUs that could be annotated to the database was 1179 (97.52%), proportion of OTUs annotated to the boundary level was 97.52%, proportion of OTUs annotated to the phylum level was 95.70%, proportion of OTUs annotated to the class level was 93.05%, and proportion of OTUs annotated to the target level was 89.00%. The proportion at the family level was 80.23%, genus level 40.03%, and species level 12.49%.

**Figure 2:**
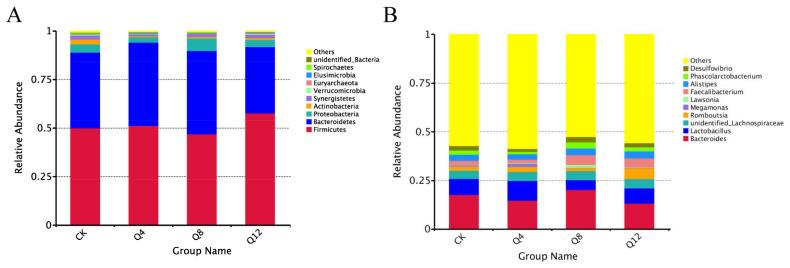
Distribution of microbial abundance. Notes: (**A**) Microbe abundance distribution at phylum level; (**B**) Abundance distribution of genus-level microorganisms.

At the phylum level (Figure 2A), *Firmicutes*, *Bacteroidetes* and *Proteobacteria* were the dominant phyla, with relative abundance greater than 3%. The relative abundance of *Firmicutes* and *Bacteroidetes* was the highest in all experimental groups, accounting for more than 89% of the total abundance. At the genus level (Figure 2B), the relative abundance of 30 genera was more than 0.1%. *Bacteroides*, *Lactobacillus* and *unidentified_Lachnospiraceae* were the dominant genera in all groups.

### 3.5. Species Diversity Analysis

**Figure 3:**
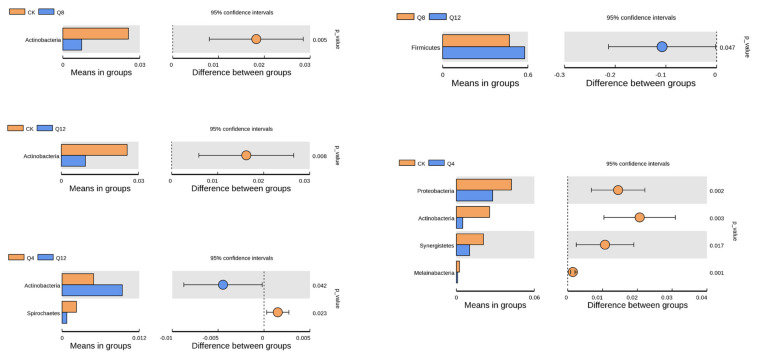
Analysis of species difference between phylum level *T*-test groups.

At the phylum level (Figure 3), compared with the experimental groups (Q4, Q8, Q12), the *Proteobacteria*, *Actinobacteria*, *Synergistetes* and *Melainabacteria* CK groups were significantly higher than those in the experimental groups (*p* < 0.05). In Q4 compared with Q12, *Actinobacteria* in the Q12 group were significantly higher than in the Q4 group (*p* < 0.05), but *Spirochaetes* in the Q12 group were significantly higher than in the Q4 group (*p* < 0.05). Compared with the Q12 group, *Firmicutes* in the Q12 group were significantly higher than in the Q8 group (*p* = 0.047).

**Figure 4:**
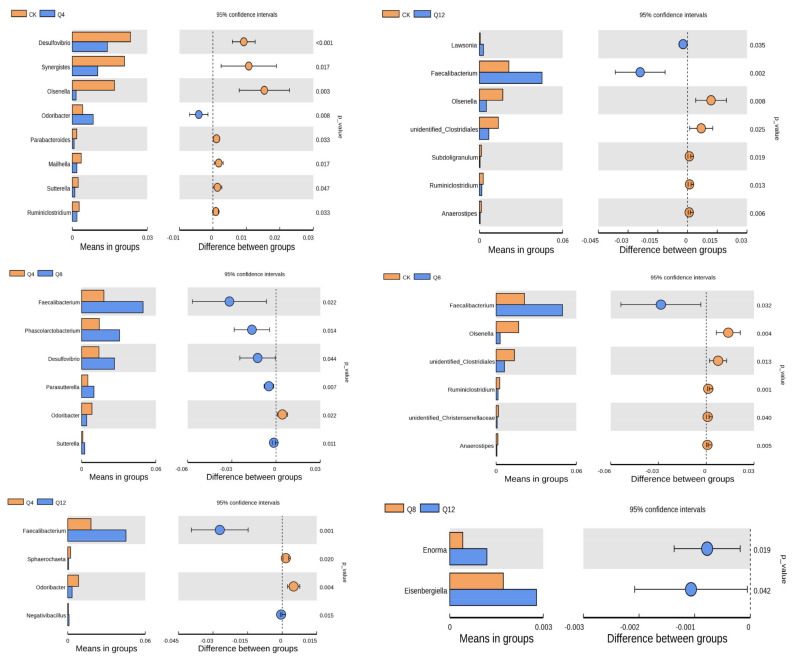
Analysis of species differences between genus level *T*-test groups.

At the genus level (Figure 4), compared with the CK group, *Desulfovibrio*, *Synergistes*, *Olsenella*, *Parabacteroides*, *Mailhella*, *Sutterella* and *Ruminiclostridium* in the CK group were significantly higher than in the group Q4 (*p* < 0.05); and *Odoribacter* bacteria in the Q4 group were significantly higher than in the CK group (*p* = 0.008). Compared with the CK group, *Faecalibacterium* in the Q8 group was significantly higher than in the CK group (*p* = 0.032). While *Olsenella*, *unidentified_Clostridiales*, *Ruminiclostridium*, *unidentified_Christensenellaceae* and *Anaerostipes* in group CK were significantly higher than in the Q8 group (*p* < 0.05). Compared with the CK group, *Lawsonia* and *Faecalibacterium* in the Q12 groups were significantly higher than in the CK group (*p* < 0.05). While *Olsenella*, *unidentified_Clostridiales*, *Subdoligranulum*, *Ruminiclostridium* and *Anaerostipes* in the CK group were significantly higher than in the Q12 group (*p* < 0.05). Compared with group Q8, *Faecalibacterium*, *Phascolarctobacterium*, *Desulfovibrio*, *Parasutterella* and *Sutterella* in group Q8 were significantly higher than in group Q4 (*p* < 0.05). While *Odoribacter* in the Q8 group was significantly lower than in the Q4 group (*p* = 0.022). Compared with the Q12 group, *Faecalibacterium* and *Negativibacillus* in the Q12 group were significantly higher than in the Q4 group (*p* < 0.05), while *Sphaerochaeta* and *Odoribacter* in the Q4 group were significantly higher than in the Q12 group (*p* < 0.05). Compared with group Q12, *Enorma* and *Eisenbergiella* in group Q8 were significantly lower than in group Q12 (*p* < 0.05).

### 3.6. Prediction of Cecal Microbial Function

**Figure 5:**
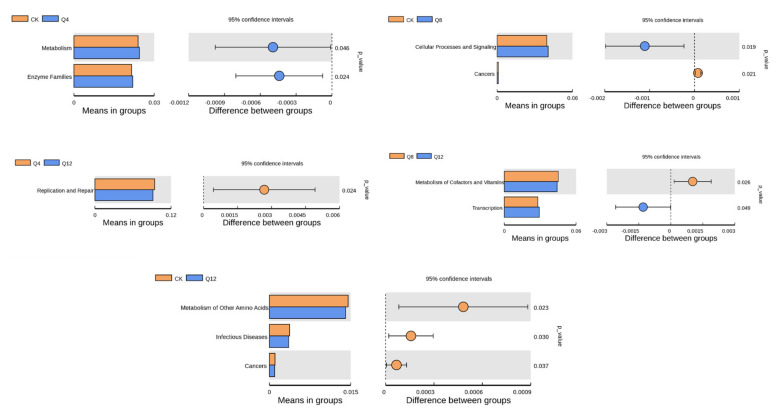
Function prediction analysis.

PICRUST was used to predict the abundance of functional genes with significant differences in the L2 level of the KEGG pathway (*n* = 6/group), and thusly analyze the functional differences between the two groups. The analysis results are shown in Figure 5. Compared with the CK group, the *Metabolism* and *Enzyme Families* pathways were significantly enriched in the Q4 group (*p* < 0.05), and *Cellular Processes and Signaling* pathways were significantly enriched in the Q8 group (*p* < 0.05), while the *Cancers*, *Metabolism of Other Amino Acids* and Infectious Diseases pathways were significantly enriched in the CK group (*p* < 0.05). Compared with group Q12, the *Replication and Repair* pathway was significantly enriched in group Q4 (*p* < 0.05) and *Metabolism of Cofactors and Vitamins* was significantly enriched in the Q8 group (*p* < 0.05), while the *Transcription* pathway was significantly enriched in the Q12 group (*p* < 0.05).

### 3.7. Correlation Analysis

**Figure 6:**
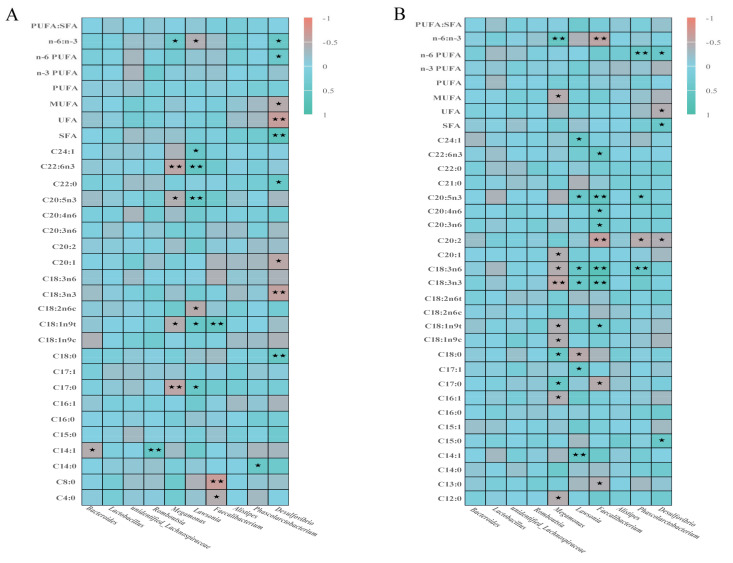
Spearman correlation analysis of caecal microorganisms and muscle fatty acids at genus level. Note: (**A**) Correlation analysis between pectoral muscle fatty acids and cecal microorganisms; (**B**) Correlation analysis between fatty acids of leg muscle and cecal microorganisms. * indicates *p* < 0.05; ** indicates *p* < 0.01.

There was a significant correlation between cecal microbiota and muscle fatty acid deposition (Figure 6), and among the fatty acids in the pectoralis muscle (Figure 6A), the abundance of *Desulfovibrio* was positively correlated with C18:0, C22:0, SFAs, n-6 PUFAs and n-6/n-3 (*p* < 0.05), and negatively correlated with C18:3n3, UFAs and MUFAs (*p* < 0.05). The abundance of *Phascolarctobacterium* was positively correlated with the abundance of C14:0 (*p* < 0.05). The abundance of *Faecalibacterium* was negatively correlated with the content of C4:0 and C8:0 (*p* < 0.05), and positively correlated with the content of C18:1n9t (*p* < 0.05). The abundance of *Lawsonia* was positively correlated with the content of C17:0, C18:1n9t, C20:5n3, C20:6n3 (*p* < 0.05), and negatively correlated with the content of n-6/n-3 (*p* < 0.05), while that of *Meagmonas* showed the opposite relationship. The abundance of *Bacteroides* was negatively correlated with C14:1 (*p* < 0.05), but *Romboutsia* showed a positive correlation.

In the fatty acids of leg muscles (Figure 6B), the abundance of *Desulfovibrio* was positively correlated with the content of C15:0, SFAs, n-6 PUFAs (*p* < 0.05), and negatively correlated with that of C20:2, UFAs (*p* < 0.05). The abundance of *Phascolarctobacterium* was significantly positively correlated with the content of C18:3n6, C20:5n3 and n-6 PUFAs (*p* < 0.05), but negatively correlated with that of C20:2 (*p* < 0.05). The abundance of *Faecalibacterium* was negatively correlated with C13:0, C17:0, C20:2 and n-6/n-3 (*p* < 0.05), and C18:1n9t, C18:3n3, C18:3n6, C20:3n6, C20:4n6, C20:5n3 and C22:6n3 were significantly positively correlated (*p* < 0.05). The abundance of *Lawsonia* was positively correlated with C14:1, C17:1, C18:3n3, C18:3n6, C20:5n3 and C24:1, but negatively correlated with C18:0. The abundance of *Meagmonas* was negatively correlated with C12:0, C16:1, C18:1n9c, C18:1n9t, C18:3n3, C18:3n6, C20:1 and MUFAs (*p* < 0.05), but positively correlated with C17:0, C18:0 and n-6/n-3(*p* < 0.05).

## 4. Discussion

### 4.1. Effect of Quinoa on Fatty Acids of Female Luhua Chicken Muscle

Studies have shown that SFAs, UFAs, PUFA/SFA, n-6/n-3 and other health indicators are important to human health, and can improve the cerebrovascular disease of the heart, and provide anti-cancer, weight loss, and immune regulation [43]. In this study, the content of SFAs in experimental groups was significantly lower than that in the CK group, while the content of UFAs showed the opposite relationship. Because of muscle fatty acid involvement in the Maillard reaction, the content of flavor compounds increases to a certain extent, thus increasing the meat flavor [44]. The richer the composition and content of UFAs in muscles, the higher and more balanced the nutritional value of the muscles. For humans, chicken is one of the major sources of UFAs, especially n-3 PUFAs, whose fatty acid composition is regulated by changing the level of fat intake and absorption [45,46]. In this study, the content of n-3 PUFAs in all experimental groups was significantly higher than in the CK group, because quinoa seeds are rich in protein, amino acids, UFAs, vitamins and mineral elements [47], among which the content of UFAs is twice that of corn kernels, and most of the UFAs are n-3 and n-6 series [48]. Therefore, adding a certain amount of quinoa in the diet can effectively improve the composition of PUFAs in the diet to a certain extent, increase the content of n-3 PUFAs in the diet, and thus improve the content of n-3 PUFAs in the muscle.

In this study, the content of C18:1n9c in pectoralis and leg muscles was the highest, followed by C16:0 and C18:2n6c. The content of C16:0 in experimental groups was significantly lower than in the CK group. C18:1n9c, C18:2n6c, C18:3n3 and DHA were significantly higher than in the CK group. This is mainly because the main SFAs found in quinoa are C16:0, and the UFAs are C18:1n9c, C18:2n6c and C18:3n3 [48,49,50]. Adding a certain proportion of quinoa in the diet changes the composition and content of fatty acids in the diet, which has a certain effect on meat quality. Meanwhile, studies have found that C18:2n6c can be metabolized to C20:4n6, and C18:3n3 can be metabolized to EPA and DHA, which play an important role in the prevention and treatment of prostaglandins, thrombosis, atherosclerosis, immunity, and anti-inflammatory and membrane function [7,51]. The results showed that the addition of quinoa to the diet promoted the deposition of PUFAs in muscle tissue, presumably because the tannin in quinoa inhibited the activity of hydrogenated microorganisms and thus prevented some of the PUFAs from being hydrogenated, and thus more PUFAs are deposited in the meat. Additionally, α-tocopherols (such as vitamin E) found in quinoa act as natural oxidants at the cell membrane level to protect fatty acids from free radical damage [48]. Among them, the World Health Organization (WHO) recommends an n-6/n-3 ratio in the range of (5–10):1 [52,53]. While the recommended range of the n-6/n-3 ratio in the Reference Intake of Dietary Nutrients for Chinese Residents is (4–6) [44]. In this study, the content of SFAs and the n-6/n-3 ratio in the chest muscle and leg muscle of experimental groups was significantly lower than in the CK group, while the UFAs were significantly higher than in the CK group. The ratio of n-6/n-3 in each experimental group was within the suitable range recommended by WHO. It showed that adding a different proportion of quinoa in the diet could improve the composition and content of fatty acids in the muscle of Luhua chickens. Studies have shown that fatty acid composition of feed is an important determinant of fatty acid composition in broiler muscle [54]. Dietary protein level or n-3 PUFAs content can affect the content and flavor of UFAs in the muscle [55,56]. Therefore, increasing dietary raw materials rich in PUFAs can change muscle fatty acid synthase (FAS) and the lipid profile [57,58] by decreasing the content of SFAs and increasing the content of UFAs in muscle [59]. This is consistent with the results of this experiment, which verifies the regulation and influence of quinoa on muscle fat. The PUFA/SFA ratio is also an important index to measure the nutritional value of muscle, and the appropriate ratio recommended by the WHO should be greater than 0.4 [52,53,54]. In this study, the ratio was about 0.54 in the pectoral muscle tissue and 0.6 in the leg muscle between the groups, both greater than 0.4, which was in line with the range recommended by the WHO.

### 4.2. Effect of Quinoa on Cecal Microbiota of Female Luhua Chickens

The poultry gut is a complex ecosystem. As the largest digestive organ for nutrient absorption and utilization, it contains a complex and highly diverse microbial community. In this study, it was found that the Simpson index of microbial diversity had some differences among groups, and the Shannon index of the CK group was significantly higher than that of other experimental groups, indicating that adding a certain proportion of quinoa in the diet had a certain effect on the cecal microbial species diversity of Luhua chickens. Studies have shown that gut microbiota is closely related to maternal nutrient level [60]. Different proportions of quinoa were added to the diet, resulting in certain differences in cecal microbial species composition.

In this study, *Firmicutes*, *Bacteroidetes* and *Proteobacteria* were the dominant phyla at the phylum level, with their relative abundance greater than 3%. The relative abundance of *Firmicutes* and *Bacteroidetes* was the highest in all experimental groups. *Firmicutes* and *Bacteroidetes* play important roles in energy metabolism in animals [61], and *Firmicutes* can produce various digestive enzymes to promote the digestion and absorption of nutrients [62]. *Bacteroidetes* showed strong degradation of protein and carbohydrates [63,64,65]. The greater the ratio of *Firmicutes: bacteroidetes* in the gut, the greater the absorption capacity of the body for energy-related substances. The smaller the ratio, the more prone the animal to become obese [63,66]. *Bacteroidetes* and *Firmicutes* constitute most of the microbial communities at the level of intestinal microbiota in broilers. The results of this study are consistent with that. The relative abundance of *Firmicutes* and *Bacteroidetes* is the highest in all experimental groups, accounting for more than 89% of the total abundance. At the genus level, the relative abundance of 30 bacterial genera detected in this study was greater than 0.1%, and *Bacteroides*, *Lactobacillus* and *unidentified_Lachnospiraceae* were all the dominant genera in each experimental group. *Bacteroidetes* have been found to improve the intestinal environment of animals, degrade plant polysaccharides, regulate electrolyte balance and improve intestinal metabolic function [67,68,69,70]. *Bacteroides* in the Q8 group were significantly higher than in the other groups, indicating that 8% quinoa added in the basal diet was conducive to the fermentation of *Bacteroides* in ceca; production of acetate, propionate and other short-chain fatty acids; and maintenance of intestinal health homeostasis. *Firmicutes* represent the largest population of gut microbes in mice and humans [71], and many species of this phylum are capable of producing endospores that are resistant to drying and other harsh conditions [72]. *Firmicutes* have been shown to be involved in energy absorption and may be associated with the development of diabetes and obesity in humans [66,73,74]. Our results are consistent with these previous studies, suggesting that a significant increase in *firmicutes* may contribute to an improved intestinal environment and healthy growth in broilers. Using PICRUST software to predict KEGG function, it was found that *Cancers*, *Infectious Diseases* and other harmful metabolic pathways were mainly enriched in the CK group. The results indicated that quinoa of different proportions in the diet could improve the structure of cecal microflora, inhibit the number of harmful microflorae, increase the number of beneficial microflora, significantly regulate the intestinal environment and promote the health of broilers. While *Metabolism* and *Enzyme Families* were significantly enriched in the Q4 group, *Cellular Processes and Signaling* were significantly enriched in the Q8 group, and *Transcription* was significantly enriched in the Q12 group. This may be because quinoa has medicinal properties such as anti-cancer, weight loss, and prevention of cardiovascular and cerebrovascular diseases [48]. After the body ingests quinoa, it affects the composition and structure of intestinal microbes through digestion and absorption, thus exerting its efficacy and having an impact on the body. Additionally, we speculate that quinoa seeds contain rich and comprehensive nutritional value, and are rich in protein, amino acids, UFAs, vitamins and mineral elements [47]. Moreover, it is rich in n-3 PUFAs, which can effectively increase the content of n-3 PUFAs in the diet, thereby changing the content of PUFAs and the number and structure of intestinal microbiota in broilers to some extent. But its specific regulatory mechanism needs to be further explored.

### 4.3. Correlation Analysis

Intramuscular fat deposition in animals is regulated by intestinal flora [24,25,30,31]. The results of this study are consistent with those of *Lactobacillus*, which can increase the content of various UFAs and the ratio of PUFA/SFA [75]. The main reason is that *Lactobacillus* has improved the fatty acid profile of broilers attacked by *Clostridium perfringens* [75]. The abundance of *Bacteroides* was positively correlated with the immune system, glycan biosynthesis and metabolism, and the signaling pathways related to cofactor and vitamin metabolism. The genus *Bacteroides* exhibits a high degree of host specificity, reflecting individual differences in the digestive system of host animals [76].

Studies have found that supplementation with probiotics can improve fatty acid composition in chickens [77], either by modulating gut microbiota or influencing fat deposition in broilers. In this study, there was a significant correlation between cecal microorganisms and muscle fatty acid deposition. The abundance of *Faecalibacterium* was negatively correlated with the content of C4:0 and C8:0, and positively correlated with C18:1n9t in pectoralis muscle. In leg muscle tissue, the abundance of *Faecalibacterium* was negatively correlated with C13:0, C17:0, C20:2 and n-6/n-3. And the content of C18:1n9t, C18:3n3, C18:3n6, C20:3n6, C20:4n6, C20:5n3, C22:6n3 were significantly positively correlated. Meanwhile, there was also a significant correlation between the abundance of *Lawsonia* and *Meagmonas* and the content of partially saturated and UFAs. This is mainly because *Bacteroides* and *Lactobacillus* are the main bacteria of fatty acid hydrogenation, and *Faecalibacterium* co-occurs with several members of *Bacteroidetes*, and *Bacteroidetes* may be the main bacteria of linolenic acid (C18:3n3) hydrogenation [78]. Because of *bacteroides*, *lactobacillus* contribute to the changes in abundance in the ceca and lead to changes in other bacterial genus abundance in the ceca, which indicates that there is a synergistic effect among the various microbial genera in the caecum, thus affecting muscle fatty acid deposition, leading to the change in fatty acid content in the muscle, but its exact regulatory mechanism is not clear and needs to be further explored. In this study, the content of n-3 PUFAs in the muscle tissue of each test group was significantly higher than that of the CK group, and the content of linolenic acid (C18:3n3) in the breast muscle of each test group was significantly lower than that of the CK group. Therefore, it can be concluded that this observation may be related to the decrease of the abundance of *Bacteroidetes*, resulting in the decrease in the hydrogenation of linolenic acid (C18:3n3), and thus the increase in the total content of n-3 PUFAs. The results are consistent with those of previous studies. Additionally, Yu [79], Yang [80], and Frutos [81] et al. found in their studies on finishing pigs and ruminants that there was a correlation between microbial abundance and muscle fatty acid content, and the results of this experiment were similar to their results. This is mainly because microbial activity is affected by dietary protein level, carbohydrates, starch type and active metabolites such as flavonoids, tannins and saponins, thereby regulating the effect of intestinal lipid metabolism on muscle PUFAs [79,80,81].

## 5. Conclusions

Adding quinoa seeds to diets significantly increased the content of muscle DHA, UFAs and n-3 PUFAs. The content of SAFs and the n-6/n-3 ratio were significantly reduced to provide ideal fatty acid composition and content. Thus, quinoa seeds can improve the structure of cecal microbiota, inhibit the number of harmful microbiota, increase the number of beneficial microbiota, significantly regulate the intestinal environment and promote the health of female Luhua chickens. We identified a degree of correlation between cecal microorganisms and muscle fatty acid deposition.

## Figures and Tables

**Table 1:** Composition and nutrient level of experimental diets (dry matter basis).

Items	Diets (%)			
	CK	Q4	Q8	Q12
Corn	64.00	64.00	64.00	64.00
Wheat middling	12.00	8.00	4.00	0.00
Quinoa seeds	0.00	4.00	8.00	12.00
Soybean meal	20.00	20.00	20.00	20.00
Limestone	1.20	1.20	1.20	1.20
CaHPO_4_	1.20	1.20	1.20	1.20
NaCl	0.30	0.30	0.30	0.30
1% Premix	1.00	1.00	1.00	1.00
Zeolite powder	0.30	0.30	0.30	0.30
Total	100	100	100	100
Nutrient levels				
Metabolic energy, ME (MJ/Kg)	12.24	12.32	12.40	12.48
Dry matter, DM	84.20	84.43	84.66	84.88
Crude protein, CP	16.12	16.18	16.25	16.31
Crude fiber, CF	2.47	2.60	2.74	2.88
Ether extract, EE	2.86	2.98	3.10	3.22
Calcium, Ca	0.87	0.88	0.88	0.88
Total phosphorus, TP	0.59	0.59	0.58	0.57
Available phosphorus, AP	0.20	0.20	0.20	0.20
Lysine, Lys	0.81	0.83	0.84	0.86
Methionine, Met	0.26	0.30	0.33	0.36
Methionine + Cystine, Met + Cys	0.56	0.59	0.61	0.64
Threonine, Thr	0.62	0.62	0.62	0.62
Tryptophan, Trp	0.19	0.19	0.18	0.18

Note: Premix (provided per kilogram of feed content): vitamin A, 158,000 IU; vitamin D, 44,000 IU; vitamin E, 500 IU; vitamin K, 39 mg; vitamin B_1_, 45 mg; vitamin B_2_, 125 mg; vitamin B_8_, 19 mg; vitamin B_12_, 0.24 mg; niacinamide, 790 mg; pantothenic acid, 300 mg; folic acid, 9 mg; H_2_O ≤ 8%; D-biotin, 2.9 mg; Zn, 730 mg; Fe, 1400 mg; Mn, 980 mg; Cu, 240 mg; I, 14 mg; Se, 4.8 mg; Ca, 10%; NaCl, 5~13%; total phosphorus (TP), 3.5%; choline chloride, 7000 mg. Nutrient level is calculated value.

**Table 2:** Effect of quinoa on composition and content of fatty acids in breast muscle of female Luhua chicken %.

Items	Group			
	CK	Q4	Q8	Q12
Butyric acid (C4:0)	0.44 ± 0.14 ^a^	0.40 ± 0.06 ^a^	0.18 ± 0.06 ^c^	0.29 ± 0.08 ^b^
Octanoic acid (C8:0)	0.35 ± 0.07 ^a^	0.20 ± 0.02 ^ab^	0.16 ± 0.03 ^b^	0.18 ± 0.004 ^b^
Myristic acid (C14:0)	0.69 ± 0.05 ^a^	0.50 ± 0.03 ^d^	0.64 ± 0.09 ^b^	0.56 ± 0.05 ^c^
Myristoleic acid (C14:1)	0.21 ± 0.03 ^c^	0.27 ± 0.02 ^b^	0.21 ± 0.03 ^c^	0.30 ± 0.04 ^a^
Pentadecanoic acid (C15:0)	3.04 ± 0.63	2.87 ± 0.37	2.83 ± 0.62	2.56 ± 0.65
Palmitic acid (C16:0)	26.96 ± 1.54 ^a^	24.84 ± 0.30 ^b^	24.88 ± 3.03 ^b^	25.43 ± 0.79 ^b^
Palmitoleic acid (C16:1)	4.64 ± 0.90 ^b^	5.82 ± 0.44 ^a^	5.46 ± 0.92 ^a^	5.35 ± 0.92 ^a^
Heptadecanoic acid (C17:0)	0.69 ± 0.17 ^a^	0.36 ± 0.09 ^b^	0.42 ± 0.10 ^b^	0.40 ± 0.05 ^b^
Heptadecenoic acid (C17:1)	0.56 ± 0.13 ^c^	0.83 ± 0.18 ^a^	0.69 ± 0.06 ^b^	0.67 ± 0.17 ^b^
Stearic acid (C18:0)	10.90 ± 1.11 ^a^	8.44 ± 0.30 ^b^	8.40 ± 1.21 ^b^	8.21 ± 0.63 ^b^
Oleic acid (C18:1n9c)	29.07 ± 5.50 ^b^	31.95 ± 2.16 ^a^	31.70 ± 4.22 ^a^	31.89 ± 2.32 ^a^
Elaidic acid (C18:1n9t)	0.29 ± 0.04 ^c^	0.51 ± 0.04 ^b^	0.49 ± 0.07 ^b^	1.04 ± 0.11 ^a^
Linoleic acid (C18:2n6c)	13.55 ± 1.25 ^a^	12.62 ± 1.50 ^b^	12.23 ± 1.39 ^b^	11.99 ± 1.01 ^b^
Linolenic acid (C18:3n3)	0.55 ± 0.13 ^b^	0.70 ± 0.04 ^a^	0.69 ± 0.08 ^a^	0.71 ± 0.06 ^a^
γ-linoleic acid (C18:3n6)	0.16 ± 0.05	0.17 ± 0.01	0.16 ± 0.03	0.14 ± 0.001
Eicosenoic acid (C20:1)	0.26 ± 0.03 ^c^	0.49 ± 0.02 ^a^	0.42 ± 0.04 ^b^	0.41 ± 0.03 ^b^
Eicosadienoic acid (C20:2)	0.21 ± 0.04 ^c^	0.75 ± 0.02 ^a^	0.45 ± 0.04 ^b^	0.46 ± 0.05 ^b^
Eicostrienoic acid (C20:3n6)	0.56 ± 0.17 ^b^	0.74 ± 0.05 ^a^	0.56 ± 0.13 ^b^	0.64 ± 0.07 ^b^
Arachidonic acid (AA,C20:4n6)	4.73 ± 1.40	4.24 ± 1.04	4.76 ± 1.53	5.22 ± 1.35
Eicosapentaenoic acid (EPA,C20:5n3)	0.86 ± 0.17 ^c^	1.08 ± 0.09 ^b^	1.07 ± 0.12 ^b^	1.27 ± 0.06 ^a^
Behenic acid (C22:0)	0.34 ± 0.12	0.30 ± 0.05	0.36 ± 0.07	0.35 ± 0.10
Docosahexaenoic acid (DHA, C22:6n3)	0.37 ± 0.11 ^c^	0.68 ± 0.08 ^b^	0.67 ± 0.14 ^b^	0.81 ± 0.08 ^a^
Nervonic acid (C24:1)	1.12 ± 0.26 ^b^	1.76 ± 0.16 ^a^	1.55 ± 0.41 ^a^	1.74 ± 0.35 ^a^
Saturated fatty acid (SFA)	42.18 ± 4.33 ^a^	37.84 ± 0.62 ^b^	38.55 ± 1.62 ^b^	37.79 ± 1.22 ^b^
Unsaturated fatty acid (UFA)	57.82 ± 4.33 ^b^	61.90 ± 0.88 ^a^	62.50 ± 4.65 ^a^	62.10 ± 1.29 ^a^
Monounsaturated fatty acid (MUFA)	37.93 ± 3.60 ^a^	41.36 ± 2.01 ^b^	42.47 ± 6.69 ^b^	41.21 ± 2.72 ^b^
Polyunsaturated fatty acid (PUFA)	20.22 ± 2.51	20.54 ± 1.65	20.03 ± 2.48	20.88 ± 2.09
n-3 Polyunsaturated fatty acids (n-3 PUFAs)	1.83 ± 0.23 ^c^	2.46 ± 0.19 ^b^	2.45 ± 0.24 ^b^	2.79 ± 0.12 ^a^
n-6 Polyunsaturated fatty acids (n-6 PUFAs)	20.29 ± 2.63 ^a^	17.58 ± 1.87 ^b^	17.44 ± 1.93 ^b^	17.79 ± 1.90 ^b^
n-6PUFA/n-3PUFA	9.88 ± 1.59 ^a^	7.18 ± 0.91 ^b^	6.81 ± 1.37 ^b^	6.39 ± 0.65 ^c^
PUFA/SFA	0.53 ± 0.12	0.54 ± 0.04	0.54 ± 0.06	0.55 ± 0.05

Note: ^a,b,c,d^ Values with different superscripts in the same row are significantly different (*p* < 0.05).

**Table 3:** Effect of quinoa on fatty acid composition and content in leg muscle of female Luhua chicken %.

Items	Group			
	CK	Q4	Q8	Q12
Lauric acid (C12:0)		0.04 ± 0.008	0.04 ± 003	0.04 ± 0.002
Tridecylic acid (C13:0)		0.51 ± 0.11	0.37 ± 0.02	0.46 ± 0.08
Myristic acid (C14:0)	1.25 ± 0.09 ^a^	0.63 ± 0.06 ^b^	0.63 ± 0.11 ^b^	0.65 ± 0.07 ^b^
Myristoleic acid (C14:1)	0.24 ± 0.02 ^b^	0.42 ± 0.03 ^a^	0.44 ± 0.03 ^a^	0.42 ± 0.02 ^a^
Pentadecanoic acid (C15:0)	0.10 ± 0.005	0.09 ± 0.008	0.10 ± 0.01	0.90 ± 0.01
Pentadecenic acid (C15:1)	0.20 ± 0.06 ^b^	0.35 ± 0.07 ^a^	0.35 ± 0.05 ^a^	0.36 ± 0.04 ^a^
Palmitic acid (C16:0)	27.41 ± 1.53 ^a^	25.76 ± 0.59 ^b^	26.06 ± 1.29 ^b^	26.03 ± 1.41 ^b^
Palmitoleic acid (C16:1)	5.50 ± 0.82 ^b^	7.63 ± 0.78 ^a^	7.63 ± 1.02 ^a^	7.45 ± 0.93 ^a^
Heptadecanoic acid (C17:0)	0.14 ± 0.01 ^a^	0.13 ± 0.02 ^b^	0.12 ± 0.02 ^b^	0.12 ± 0.02 ^b^
Heptadecenoic acid (C17:1)	0.13 ± 0.04 ^b^	0.28 ± 0.03 ^a^	0.28 ± 0.02 ^a^	0.26 ± 0.02 ^a^
Stearic acid (C18:0)	11.22 ± 1.55 ^a^	7.05 ± 1.55 ^b^	7.19 ± 1.02 ^b^	7.21 ± 0.61 ^b^
Oleic acid (C18:1 n9c)	29.89 ± 2.59 ^b^	34.14 ± 3.37 ^a^	33.12 ± 2.80 ^a^	34.19 ± 2.28 ^a^
Elaidic acid (C18:1n9t)	0.31 ± 0.03 ^c^	0.43 ± 0.04 ^b^	0.42 ± 0.03 ^b^	0.88 ± 0.05 ^a^
Linoleic acid (C18:2n6c)	14.37 ± 1.51 ^b^	15.44 ± 1.95 ^a^	14.93 ± 1.13 ^b^	14.31 ± 1.64 ^b^
Linolelaidic acid (C18:2n6t)	0.13 ± 0.01 ^a^	0.09 ± 0.02 ^b^	0.09 ± 0.02 ^b^	0.09 ± 0.02 ^b^
Linolenic acid (C18:3n3)	1.08 ± 0.04 ^d^	1.21 ± 0.09 ^c^	1.38 ± 0.07 ^a^	1.29 ± 0.06 ^b^
γ-linoleic acid (C18:3n6)	0.10 ± 0.03 ^d^	0.12 ± 0.02 ^c^	0.19 ± 0.04 ^a^	0.16 ± 0.03 ^b^
Eicosenoic acid (C20:1)	0.25 ± 0.04 ^b^	0.42 ± 0.04 ^a^	0.41 ± 0.05 ^a^	0.40 ± 0.03 ^a^
Eicosadienoic acid (C20:2)	0.19 ± 0.05 ^b^	0.31 ± 0.06 ^a^	0.16 ± 0.03 ^bc^	0.12 ± 0.06 ^c^
Eicostrienoic acid (C20:3n6)	0.35 ± 0.11 ^a^	0.25 ± 0.08 ^b^	0.36 ± 0.10 ^a^	0.30 ± 0.05 ^ab^
Arachidonic acid (AA,C20:4n6)	2.79 ± 0.72 ^a^	2.52 ± 0.62 ^ab^	2.18 ± 0.46 ^b^	2.48 ± 0.56 ^ab^
Eicosapentaenoic acid (EPA,C20:5n3)	0.70 ± 0.12 ^c^	0.81 ± 0.09 ^b^	0.92 ± 0.07 ^a^	0.84 ± 0.07 ^b^
Heneicosan oic acid (C21:0)	0.29 ± 0.05 ^a^	0.04 ± 0.002 ^b^	0.04 ± 0.002 ^b^	0.09 ± 0.05 ^b^
Behenic acid (C22:0)	0.24 ± 0.07 ^a^	0.15 ± 0.08 ^b^	0.15 ± 0.05 ^b^	0.20 ± 0.08 ^ab^
Docosahexaenoic acid (DHA,C22:6n3)	0.75 ± 0.10 ^c^	0.80 ± 0.04 ^b^	0.84 ± 0.03 ^a^	0.84 ± 0.06 ^a^
Nervonic acid (C24:1)	0.48 ± 0.16 ^b^	0.70 ± 0.14 ^a^	0.60 ± 0.12 ^a^	0.68 ± 0.11 ^a^
Saturated fatty acid (SFA)	42.85 ± 3.85 ^a^	34.27 ± 1.65 ^b^	34.76 ± 1.43 ^b^	34.73 ± 1.38 ^b^
Unsaturated fatty acid (UFA)	57.02 ± 4.11 ^b^	65.72 ± 1.66 ^a^	65.22 ± 1.44 ^a^	65.32 ± 1.42 ^a^
Monounsaturated fatty acids (MUFA)	36.65 ± 3.08 ^b^	44.34 ± 3.77 ^a^	43.24 ± 2.93 ^a^	44.56 ± 2.57 ^a^
Polyunsaturated fatty acids (PUFA)	19.85 ± 2.26 ^b^	21.38 ± 2.79 ^ab^	21.98 ± 2.38 ^a^	20.76 ± 2.76 ^ab^
n-3 polyunsaturated fatty acids (n-3 PUFA)	2.42 ± 0.37 ^d^	2.82 ± 0.17 ^c^	3.14 ± 0.13 ^a^	2.98 ± 0.14 ^b^
n-6 polyunsaturated fatty acids (n-6 PUFA)	17.43 ± 1.83	18.36 ± 2.73	18.69 ± 2.35	17.68 ± 2.69
n-6PUFA/n-3PUFA	8.10 ± 1.60 ^a^	6.52 ± 0.94 ^b^	5.96 ± 0.75 ^b^	5.94 ± 0.88 ^b^
PUFA/SFA	0.46 ± 0.07 ^b^	0.62 ± 0.08 ^a^	0.63 ± 0.07 ^a^	0.60 ± 0.09 ^a^

Note: ^a,b,c,d^ Values with different superscripts in the same row are significantly different (*p* < 0.05).

**Table 4:** Statistics of sample sequencing data processing results.

Items	Sample Group			
	CK	Q4	Q8	Q12
Raw PE	91,767	88,872	91,771	88,116
Raw Tags	85,365	84,815	88,060	84,872
Clean Tags	83,636	83,125	86,507	83,432
Effective Tags	63,247	66,096	67,843	65,090
Base	26,377,047	27,643,822	28,334,530	27,092,227
AvgLen	417	418.33	417.5	416
Q20	98.44	98.36	98.45	98.515
Q30	94.95	94.72	94.935	95.11
GC (%)	53.2	52.90	53.22	53.08
Effective (%)	68.77	74.38	73.98	73.95

**Table 5:** Alpha diversity indices.

Items	Group			
	CK	Q4	Q8	Q12
observed_species	640.67 ± 24.53	635.00 ± 76.96	636.50 ± 54.56	593.17 ± 47.69
Shannon	6.54 ± 0.30 ^a^	5.98 ± 0.07 ^b^	6.17 ± 0.18 ^b^	6.10 ± 0.24 ^b^
Simpson	0.97 ± 0.01 ^a^	0.95 ± 0.01 ^b^	0.96 ± 0.01 ^ab^	0.96 ± 0.01 ^ab^
chao1	668.93 ± 18.73	682.35 ± 89.63	674.81 ± 56.79	631.91 ± 52.24
ACE	675.88 ± 20.52	681.89 ± 88.39	686.06 ± 55.86	636.88 ± 48.49
PD_whole_tree	43.46 ± 5.24	43.88 ± 9.36	39.70 ± 2.49	38.58 ± 3.01

Note: ^a,b^ Values with different superscripts in the same row are significantly different (*p* < 0.05).

**Table 6:** Annotated overview of cecal microbial species.

OTU catalogue	1209	Annotated on Class level	93.05%
Annotated on Database	1179 (97.52%)	Annotated on Class level	89.00%
Annotated on Unclassified	30 (2.48%)	Annotated on Family level	80.23%
Annotated on Kingdom level	97.52%	Annotated on Genus level	40.03%
Annotated on Phylum level	95.70%	Annotated on Species level	12.49%

## Data Availability

The sequencing data were deposited into the Sequence Read Archive (SRA) of NCBI (PRJNA905337).
